# 肺癌合并慢性阻塞性肺疾病患者围手术期气道管理现状

**DOI:** 10.3779/j.issn.1009-3419.2014.12.11

**Published:** 2014-12-20

**Authors:** 国卫 车, 修益 支

**Affiliations:** 1 610041 成都，四川大学华西医院胸外科 Department of Thoracic Surgery, West China Hospital, Sichuan University, Chengdu 610041, China; 2 100053 北京，首都医科大学宣武医院胸外科 Department of Thoracic Surgery, Xuanwu Hospital, Capital Medical University, Beijing 100053, China

**Keywords:** 围手术期气道管理, 慢性阻塞性肺疾病, 肺肿瘤, Perioperation airway management, Chronic obstructive pulmonary disease, Lung neoplasms

## Abstract

肺癌患者均合并不同程度的慢性阻塞性肺疾病（chronic obstructive pulmonary disease, COPD），而COPD导致的肺功能降低对其能否手术治疗及术后并发症发生具有重要的影响。研究证明围手术期气道管理可以有效改善患者肺功能且减少术后并发症。本文针对近年来气道管理的临床应用现状及进展进行综述，主要有以下几方面：①围手术期气道管理的必要性；②围手术期气道管理的药物治疗现状与特点；③围手术期应用气道管理在改善肺功能中的价值；④围手术期需要气道管理的最佳人群；⑤围手术期气道管理应用存在的问题。

## Abstract (en)

The close association between chronic obstructive pulmonary disease (COPD) and lung cancer has long been known. New evidence suggests that there is an inverse relationship between the severity of pulmonary function and the risk of surgery in patients with lung cancer, COPD may play a key role in the incidence of postoperative pulmonary complications (PPC) and improving pulmonary function with perioperation airway management has been suggested as a possible preventive PPC. This review describes the current understanding and clinical application of perioperation airway management in lung cancer patients with COPD. We summarized that: (1) The necessity of perioperation airway management; (2) The status and characteristic of inhaled drug in perioperation airway management; (3) The clinical value in improving pulmonary function of perioperation airway management; (4) The optimum population of peri-operation airway management; (5) The problem of perioperation airway management.

肺癌的主要治疗手段仍是外科手术治疗，而患者同时合并慢性阻塞性肺病（chronic obstructive pulmonary diseases, COPD）是术后并发症增加和失去根治性手术切除机会的主要因素。术后的主要并发症是呼吸相关并发症（postoperative pulmonary complications, PPC）（如：肺部感染等）及其导致的死亡^[1]^。研究表明术后发生PPC的患者围手术期多有不同程度的COPD^[2]^，围手术期针对肺癌合并COPD患者的气道管理（物理治疗或药物治疗），不但可以降低术后PPC且也能使更多的患者获得手术机会^[3, 4]^。围手术期气道管理目前主要集中在雾化吸入药物（糖皮质激素或支气管扩张剂）能否在短期内（2周内）改善肺功能及降低术后呼吸道并发症的研究，且已取得了明显的效果。本文主要就围手术期气道管理现状和进展进行综述。

## 1 围手术期气道管理的必要性

当前新诊断的肺癌患者中有40%-70%合并有不同程度的COPD^[5]^，且已戒烟的肺癌合并COPD患者行外科治疗的风险是无COPD的6倍^[6]^，而COPD是术后并发症和死亡率的独立预后因子^[4]^。Smetana等^[7]^通过分析发现当前预防术后肺部并发症的必要性有：①非心脏手术的术后肺部并发症（2.7%）与心脏并发症发病率（2.5%）相当；②呼吸衰竭是疾病治疗过程中病情恶化和诱发新并发症的主要原因，术后若发生呼吸衰竭则患者30天内死亡率为26%，6%的患者发生心肌梗塞，35%的发生肺炎，10%的发生急性肾衰，3%产生深静脉栓塞和肺栓塞，而没有发生呼吸衰竭的患者发生各种并发症的几率小于2%；③术后并发症必然导致医疗成本的增加和住院时间表延长。另外基于当前空气污染和吸烟仍将是长时期存在的致肺癌的主要危险因素，由此导致的气道损伤和肺功能降低也是肺癌患者失去外科治疗机会和增加手术风险的主要因素。肺癌的外科治疗是患者延长生命和获得治愈的主要手段，目前仍是不可替代的治疗方案。吸入性药物（支气管扩张剂和糖皮质激素）在治疗或预防呼吸系统相关疾病（哮喘或COPD）中取得明显的效果，且有研究^[8]^发现围手术期合理应用吸入性药物可以有效降低术后哮喘发作或COPD的加重。同时，围手术期吸入性药物治疗和肺康复训练可以有效改善肺癌患者的肺功能且促进术后快速康复。因此，肺癌合并COPD患者围手术期气道管理成为当前的研究热点和必然。

## 2 围手术期气道管理药物治疗现状与特点

从PUBMED数据库（时间截止至2014年7月7日）分析应用三种不同药理作用的吸入性药物治疗呼吸系统疾病（主要是肺癌、慢性阻塞性肺疾病和哮喘）发表文章数量可以总结出这样的共识：①Budenoside（布地奈德）和Formoterol（福莫特罗）临床应用和研究最多的是哮喘，且布地奈德是福莫特罗的1倍多；Tiotropium（噻托溴胺）则主要用于治疗COPD（图 1）。②布地奈德关于肺癌的研究，发表文章有41篇，基础研究31篇，10篇临床研究中也有8篇集中在布地奈德对抑制肺癌生长作用的研究；肺癌患者围手术期应用布地奈德的研究有2篇，1篇是肺癌患者术中麻醉，单肺通气应用布地奈德可以降低气道阻力^[9]^，仅有1篇是肺癌术前用药可以改善肺功能^[10]^。③噻托溴胺在肺癌中的研究均集中在合并COPD患者行肺叶切除术患者术前准备，肺功能改善和降低术后并发症方面，且均证实术前应用噻托溴胺治疗可以降低术后并发症且改善肺功能；福莫特罗单独应用于肺癌的研究尚未见报道。④从发表文章的年代和种类看，COPD的主要治疗药物目前仍以噻托溴胺为主，但2010年以后布地奈德用于COPD的研究的文章增长最快，而应用于哮喘的研究在减少（图 2，图 3）；将吸入性药物应用于肺癌合并COPD需要手术患者的围手术期的治疗，是研究的方向。但也存在以下问题：①三种药物的临床研究中均存在回顾性文章多，2篇随机研究中也存在研究样本含量少且是单中心的研究。②研究肺功能改善的文章多，而对术后并发症研究的少，外科临床应用证据少。③现有研究中也存在药物种类、每种药物的剂量、持续时间均不同，缺乏统一的标准，临床应用指导价值差。这些文章均分析产生这些问题的原因是胸外科医生对围手术期气道管理和肺功能保护的重视程度不够，肺癌合并COPD患者术后发生严重PPCs的比例较低，且忽视了术后患者的生活质量。

**1:**
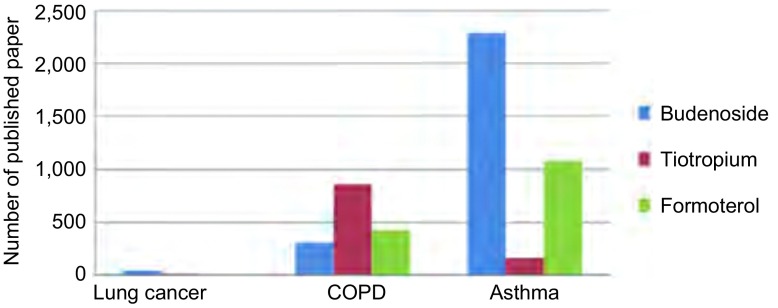
三种药物关于肺疾病发表文章分析 The published articles of lung disease on inhaled drugs. COPD: chronic obstructive pulmonary disease.

**2:**
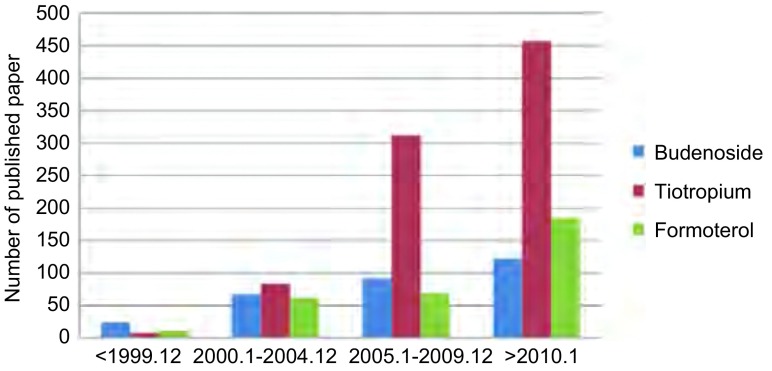
三种药物关于COPD研究发表文章分析 The published articles of COPD on inhaled drugs

**3:**
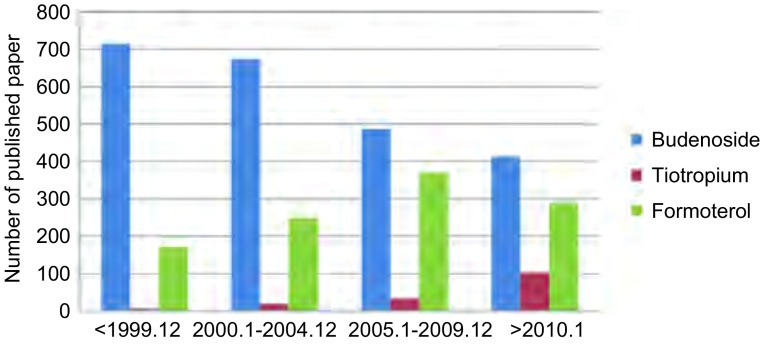
三种药物关于哮喘发表文章分析 The published articles of asthama on inhaled drugs

## 3 围手术期气道管理在改善肺功能中的作用

研究^[11, 12]^表明：空气污染、长期吸烟或内源性因素导致的COPD患者，均存在不同程度的小气道痉挛持续状态，不仅导致气道阻力增高，也使痰液难以排出，同时气道长期存在炎症刺激而使呼吸膜增厚，既有通气障碍也有换气问题，尤其是肺癌合并COPD患者的肺功能下降更明显。因此，术前对气道管理理论上应该能改善肺功能，Suzuki等^[13]^前瞻性研究了20例肺癌合并中-重度COPD患者行肺叶切除术患者，实验组（10例）围手术期每天应用（tiotropium和salmeterol），对照组（10例），结果表明：治疗组的第1秒用力呼气容积（forced expiratory volume in one second, FEV1）、用力肺活量（forced vital capacity, FVC）、IC（inspiratory capacity）和膈肌运动能力（diaphragmatic motion）均优于对照组（*P* < 0.05）。Ueda等^[14]^前瞻性地收集了44例肺癌合并COPD患者的手术资料，其中24例围手术期没有应用tiotropium治疗，20例应用，结果提示：Tiotropium治疗组术前肺功能指标均得到明显改善。而术后应用吸入性支气管扩张剂也可改善肺功能，且提高生活质量。Nojiri等^[15]^研究了21例肺癌合并COPD行肺叶切除术患者，术后进行3个月的tiotropium吸入治疗，发现FVC变化无统计学差异，而FEV1明显改善（1.60 L±0.5 L *vs* 1.84 L±0.5 L）（*P* < 0.001）。Kobayashi等^[16]^也回顾性分析了102例肺癌合并COPD的患者术前2周应用噻托溴胺治疗，发现治疗前后其呼吸道症状和肺功能均明显改善，如FVC（均值治疗前3.43 L *vs*治疗后3.52 L），FEV(1)（均值2.06 L *vs* 2.32 L）和FEV(1)%（73.2% *vs* 81.0%）（*P* < 0.001）；术后FEV(1)%从56.0%（51.6%-60.3%）增加到63.4%（60.8%-66.0）（*P* < 0.001）；FEV1增加值与COPD严重程度呈负相关（*r*=-0.59, *P* < 0.005），术后FEV1的绝对值在tiotropium治疗组预测值1.65 L明显低于实测值1.96 L（*P* < 0.001）。从理论上讲单独应用支气管扩张剂可以有效改善气道痉挛，改善通气提高肺泡内氧分压，但对改善换气和气道炎症控制效果差或没有。而糖皮质激素类吸入性药物（如布地奈德）不但可以缓解小气道痉挛，也可以通过消除气道炎症和呼吸膜水肿而改善换气功能，同时也助于气道内环境的改善。Bolukbas等^[10]^对肺癌合并中-重度COPD患者行肺叶切除术患者术前联合应用噻托溴胺/福莫特罗/布地奈德和噻托溴胺/福莫特罗方案研究表明，前者对FEV1的绝对值增加程度，FEV1增加10%患者数和术后并发症发生率均优于后者，提示我们将布地奈德应用于COPD的治疗有其独特的优势。当然围手术期气道管理也可降低术后并发症，Nojiri等^[17]^回顾性分析了104例肺癌合并中-重度COPD患者行肺叶切除术患者围手术期应用和不用噻托溴胺进行研究发现，治疗组的术后心肺并发症明显低于对照组（18% *vs* 48%, *P*=0.001），且治疗组患者术后白细胞计数和C反应蛋白水平也明显降低。恰当的围手术期气道管理也可为高风险的肺癌患者提供手术机会，Matsuyama等^[18]^对3例肺癌合并严重COPD的患者（FEV1 < 40%）进行为期术前2周-4周的tiotropium bromide治疗，患者肺功能均得到明显改善且安全进行肺叶切除术，术后1年随访肿瘤没有复发且肺功能好，提示术前应用tiotropiumbromide可以改善肺功能并为患者提供手术机会。

## 4 围手术期需要气道管理的最佳人群

目前国际上关于肺癌合并COPD患者围手术期气道管理或肺功能保护的研究多集中在支气管扩张剂或糖皮质激素雾化治疗上，其实药物治疗只是肺康复（pulmonary rehabilitation, PR）的一个方面。事实上COPD患者术后肺部并发症发生的主要原因是痰液分泌增加和排出障碍，药物处理可以减少痰液分泌，而物理康复（呼吸训练）则可以增加患者运动耐力使痰液更容易排出^[19]^。因此将药物与物理康复结合起来的肺康复方案（pulmonary rehabilitation program, PRP）将是最佳的治疗方法。但是也不是所有的肺癌均需要术前的肺康复训练，只有合并高危因素（如COPD等）的肺癌患者才有进行康复的必要。结合现有临床研究^[20-22]^和心肺运动试验（cardiopulmonary exercise test, CPET）和静态肺功能（resting pulmonary function test, PFT）检查发现的肺癌患者术前高危因素有：①支气管高反应性；发生率为19.88%（68/342）^[4]^；②峰值呼气流量（peak expiratory flow, PEF）PEF < 250 L/min，发生率13.74%（47/342）^[4]^；③肺功能处于临界状态（1.0 L < FEV1 < 1.2 L，且40% < FEV1% < 60%）^[4]^；④术前吸烟时间大于800年支且戒烟时间小于2周^[4]^；⑤术前气管内定植菌存在^[2]^。同时对这些高危因素进行的患者进行术前的肺康复训练（物理训练+药物治疗），发现康复组患者术后并发症和肺部感染发生率均较未康复组下降5倍^[4]^。进一步研究肺功能差不能手术的肺癌患者进行肺康复训练2周，肺功能达到可手术的标准，且术后的并发症未较同期手术的并发症增加^[3]^。通过对手术前后肺癌合并COPD患者心率和血氧饱和度及运动耐力的研究发现，术前肺康复训练可以有效改善患者的生活质量^[23, 24]^。

## 5 围手术期气道管理应用存在的问题及研究方向

围手术期气道管理的临床应用，从胸外科围手术期气道管理专家共识（2012年版）发布以来^[25]^，得到大家的重视且也取得了良好的效果，且已取得以下共识：①可以降低肺癌患者术后并发症，尤其是肺部并发症；②为因肺功能差而不能手术的肺癌患者提供手术机会；③改善肺癌患者术后的生活质量。但无论从临床应用到研究仍存在以下问题：①各医疗中心应用中的方案不统一，且多是经验应用，缺乏临床证据；②所有的研究均是单中心单个医疗组连续入组患者，非随机研究，以观察为主；③研究样本含量小，且手术方式不统一，如：胸腔镜肺叶切除术和开胸手术未区分研究；④术前肺康复方案不统一，尤其是药物康复（用药种类和课题差异大）；⑤没有对肺功能改善及并发症降低的机制进行研究。因此，进行多中心、大样本围手术期气道管理和肺康复训练的临床研究非常必要且刻不容缓。
